# Young People’s Knowledge of Antibiotics and Vaccinations and Increasing This Knowledge Through Gaming: Mixed-Methods Study Using e-Bug

**DOI:** 10.2196/10915

**Published:** 2019-02-01

**Authors:** Charlotte Victoria Eley, Vicki Louise Young, Catherine Victoria Hayes, Neville Q Verlander, Cliodna Ann Miriam McNulty

**Affiliations:** 1 Primary Care Unit Public Health England Gloucester United Kingdom; 2 Statistics Unit Public Health England London United Kingdom

**Keywords:** education, children, knowledge, antibiotics, vaccines

## Abstract

**Background:**

e-Bug, led by Public Health England, educates young people about important topics: microbes, infection prevention, and antibiotics. Body Busters and Stop the Spread are 2 new e-Bug educational games.

**Objective:**

This study aimed to determine students’ baseline knowledge, views on the games, and knowledge improvement.

**Methods:**

Students in 5 UK educational provisions were observed playing 2 e-Bug games. Before and after knowledge and evaluation questionnaires were completed, and student focus groups were conducted.

**Results:**

A total of 123 junior and 350 senior students completed the questionnaires. Vaccination baseline knowledge was high. Knowledge increased significantly about antibiotic use, appropriate sneezing behaviors, and vaccinations. In total, 26 student focus groups were conducted. Body Busters was *engaging* and *enjoyable*, whereas Stop the Spread was *fast-paced* and *challenging* but increased vaccination and health behavior intentions.

**Conclusions:**

e-Bug games are an effective learning tool for students to enhance knowledge about microbes, infection prevention, and antibiotics. Game-suggested improvements should help increase enjoyment.

## Introduction

Educating children and young people is important in the fight against antibiotic resistance. Through education, we can raise awareness, enhance knowledge, and modify behavioral intentions about hygiene and antibiotic use in our future generation of antibiotic users. E-Bug, led by Public Health England (PHE), is an international health education resource that teaches children and young people about hygiene, the spread of infections, antibiotic use, and resistance. e-Bug includes Web-based lesson plans and activities for educators and educational games for students hosted on the e-Bug website. Evaluation of e-Bug activities to be undertaken in schools and science shows has been well-documented [1-6], and the National Institute of Clinical Excellence [7] has suggested that schools may use the evidence-based e-Bug resources to educate children and young people in an age-appropriate way about hygiene, prevention of infections, and antibiotic use.

Google Analytics has been used to monitor Web traffic to the e-Bug website since 2010 [2]. The e-Bug website had 94,675 visitors from September 1, 2016, to August 31, 2017, and 100,955 visitors in the previous academic year. The e-Bug games’ home page was the second highest visited page with 28,610 page views from September 1, 2016, to August 31, 2017; the senior students games’ home page had 10,154 views during the same period.

The internet is a suitable tool for health promotion, and internet-based health interventions have been shown to change health behaviors. *Gamification*, where features of gaming are used in other disciplines, has become increasingly popular in recent years, aiming to make science and health education more available and exciting to the general public. *Serious games* are those games where the primary focus is not entertainment but education and learning [8]. A meta-analysis of serious games in regard to their effect on cognitive processes and motivation found the games to positively affect cognitive processes, including learning and retention compared with traditional educational methods, with no difference to motivation [9].

Evidence suggests that gamification and serious games for health and well-being are most effective when targeting health-related behaviors [10]. For instance, positive associations between gamification, serious games, and school-aged knowledge and behavior have been reported in public health topics such as asthma [11], fruit and vegetable consumption [12], and oral hygiene [13,14].

The e-Bug Web-based educational games [15] have been previously evaluated including a study that evaluated 3 e-Bug games using a mixed-methods approach; the 3 e-Bug games showed an improvement in knowledge and focused on the use of antibiotics for bacterial versus viral infections and ensured that the course of antibiotics is completed [16].

The aim of this study was to evaluate the 2 e-Bug educational games: Body Busters, previously evaluated by Hale et al and then modified with new content [16], and Stop the Spread, a new educational game launched in 2016. The questions of the research study are as follows:

- What is students’ baseline knowledge about the game-learning outcomes?
- What is students’ change in knowledge following the games?
- What are students’ views on the games to suggest improvements?

**Figure 1:**
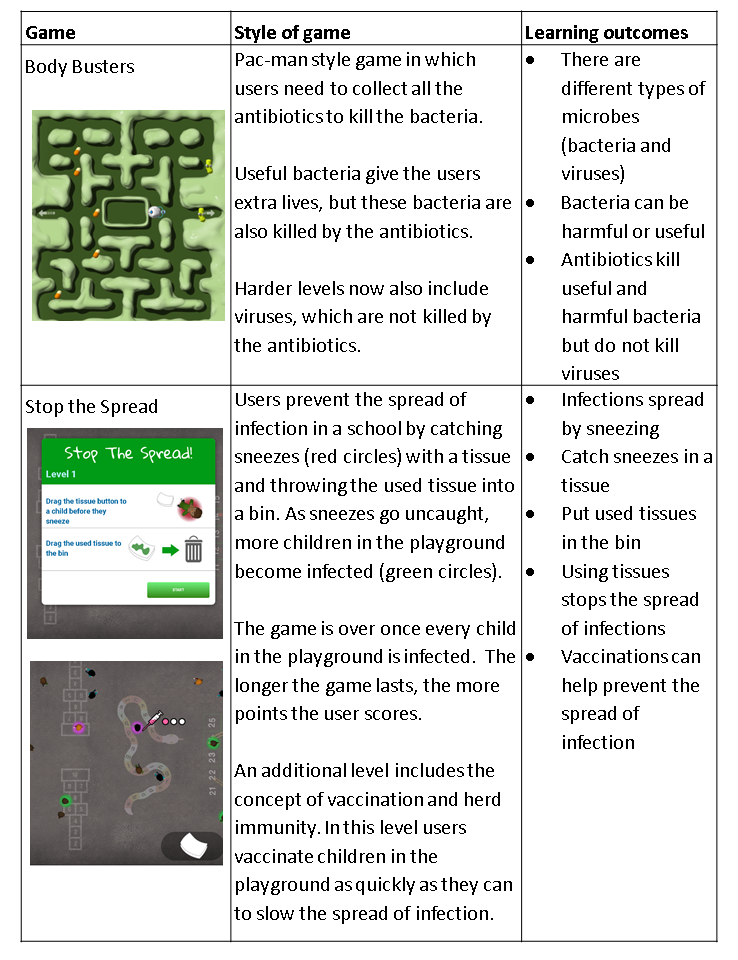
The style of play and learning outcomes of Body Busters and Stop the Spread.

Figure 1 details the style of play and learning outcomes of the 2 games. Both e-Bug games are responsive on all devices including computers and tablets. Pilot game testing was conducted at 3 schools before the game launch to ensure the games worked correctly and the instructions were clear.

## Methods

### Research Design

The study was a mixed-method evaluation using quantitative and qualitative methods. Quantitative methods included before and after students’ knowledge questionnaires; qualitative methods included students’ focus groups and open-ended questions and responses from the students’ postgaming evaluation questionnaire.

### Sampling and Recruitment

Educational providers, including schools and summer schools, were invited to take part in the study through convenience recruitment of educators at educational and scientific conferences and then through snowball sampling. Sampling aimed to ensure a representation of school-aged children across 3 local authorities in the United Kingdom, including rural and urban schools, different socioeconomics, and selective grammar and nonselective state schools (see Table 1). Local authorities were Gloucestershire, Buckinghamshire, and South Wales.

### Ethics

All researchers who observed the sessions had a Disclosure Barring Check, through PHE, to work with children. This study did not require National Research Ethics Service approval as it was outside the National Health Service and was classed as a service evaluation. PHE provided written confirmation approving the service evaluation in July 2016. Educational providers gave informed written consent before the study took place; students were involved and their parents were given the option for students to opt out at any point during data collection. Teachers reported that no students opted out of the research. Consent was deemed accepted if the participants completed the before and after knowledge questionnaires. Questionnaires were collected in line with the Data Protection Act 1998 and Caldicott 1999 regulations on handling and distributing sensitive participant information. Focus group participants provided verbal informed consent for participation in the study, audio recording, and the publishing of anonymized quotes.

### Data Collection and Analysis

Data collection took place between August 2016 and July 2017.

#### Quantitative Data

Before and after knowledge questionnaires were used to evaluate whether playing the e-Bug games had made any change to students’ knowledge. Questionnaires were based on previously validated questionnaires used to evaluate the e-Bug games and activities [2,3,16]; 4 additional questions (1, 2, 3, and 5) were included in the questionnaire to cover additional learning outcomes that could be indirectly improved through game play and are hereby referred to as *general questions* (see Multimedia Appendix 1 for questionnaires).

Data collection consisted of (1) students completed pregame-play questionnaire 1 alone without consultation, (2) students played on Body Busters for 5 min, (3) students played on Stop the Spread for 5 min, and (4) students completed postgame-play questionnaire 2 alone without consultation, which also included some additional open-ended evaluation questions. The game play was for 5 min to follow the methodology of previous evaluations [16], and as 5 min is the estimated amount of time it takes to play 1 round of the games from testing, it allowed the study to measure knowledge change after single game play rather than repeated game play.

**Figure 2:**
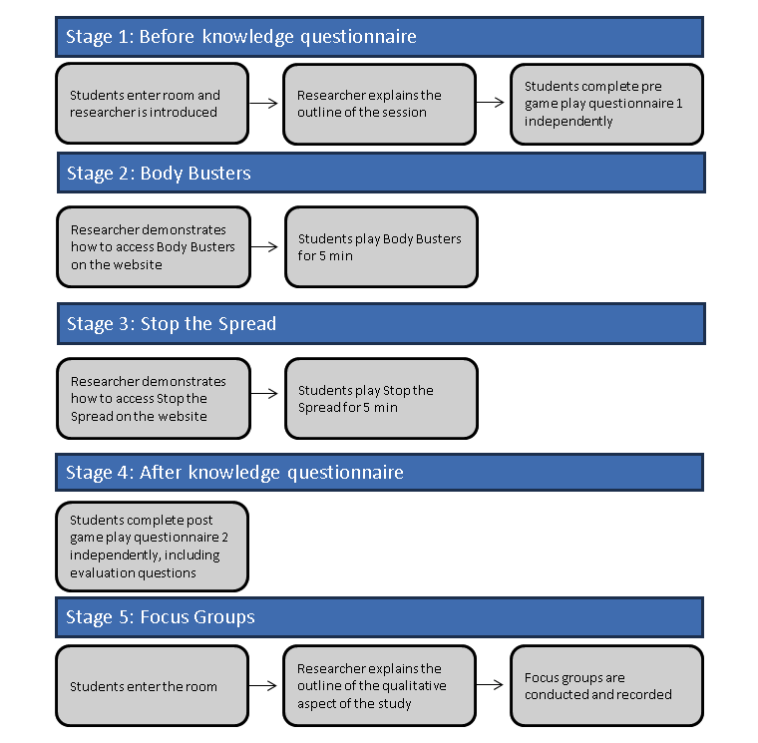
e-Bug game play and data collection process.

A researcher was present in each session to monitor and observe game play and hand out and collect questionnaires from the students. Data collection occurred in a convenient room where students had their own computer. The rationale for that was to model how the games might be played in a real-life teaching situation. Figure 2 provides further details on the data collection process.

McNemar test was used for each response from the multiple-choice question to determine the significance of the difference in the proportion of correct answers before and after game playing. Moreover, 95% CIs of the odds ratio were estimated to determine the odds of students answering correctly. Analysis was performed separately for junior and senior school-aged pupils, as knowledge change could differ between age groups. All statistical analysis was completed in STATA, version 14.2.

The postgame-play questionnaire 2 included an additional 7 questions on game enjoyment, including 2 Likert scale questions (students circled a number scale of 1-10) and 5 open-ended questions. Likert scale responses were inputted into MS Excel, and mean enjoyment scores for each game were calculated for junior, senior, and all students.

**Table 1:** Demographics of educational providers.

Educational provider	Local authority	Type of education	Questionnaires (N=473), n (%)	Focus groups (N=26), n (%)	Students (N=126), n (%)
A	Gloucestershire	Summer school	61 (13)	14 (54)	61 (48)
B	Gloucestershire	Grammar	29 (6)	1 (4)	6 (5)
C	South Wales	State	100 (21)	4 (15)	24 (19)
D	Bedfordshire	State	183 (39)	4 (15)	20 (16)
E	Bedfordshire	Grammar	100 (21)	3 (12)	15 (12)

#### Qualitative Data

In total, 5 open-ended questions on enjoyment were included in the postgame-play questionnaire 2 to provide deeper qualitative data from all students.

Semistructured focus groups were facilitated immediately after the game intervention by VLY, CVH, and CVE who are all trained qualitative researchers for the e-Bug project, PHE. All 26 focus groups took place in person in a convenient room at the educational establishment. Focus groups of size 4 to 6 students, chosen by the class teacher and represented a mix of student abilities, lasted for 6 to 20 min depending on participant age. Focus groups were audio recorded, transcribed verbatim, and checked for accuracy by CVH or CVE. Furthermore, 26 focus groups were planned with all 5 schools participating; no new themes emerged from the later focus groups, and researchers agreed that data saturation had been reached.

The topic guide for the focus groups was based on previous e-Bug evaluation topic guides for e-Bug and included additional questions on Stop the Spread learning outcomes. The schedule was piloted during the e-Bug game development in 3 testing sessions in schools in March 2016.

All focus group data and open-ended responses on enjoyment were inputted into NVivo 10 (QSR International) qualitative analysis software. NVivo 10 was used to organize, code, and analyze the focus group transcripts by CVH and open-ended evaluation responses by CVE. A subset of focus group data (2 junior and 2 senior transcripts) was analyzed by a second researcher (CVE) to ensure reliability. Both researchers discussed the data and coding to agree on the emerging themes before developing a thematic framework. Any discrepancies between researchers were resolved through discussion until an agreement was reached. The thematic framework was discussed by the research team.

## Results

### Main Findings

The study recruited 473 students (123 junior and 350 senior students) aged 7 to 16 years from 5 educational providers across 3 local authorities in the United Kingdom (illustrated in Table 1).

Before and after knowledge questionnaires were completed by 473 students. Baseline knowledge about vaccinations was high in junior students (>60% correct responses) and was higher in senior students (>80% correct responses), except for 1 question for which the baseline knowledge 55%. Baseline knowledge about antibiotics was low in junior and senior students (<40% and <67% correct responses, respectively). Senior students had greater pregaming knowledge than junior students in 11 out of the 12 questions and had greater postgaming knowledge in 10 out of the 12 questions. However, knowledge change was greater in 9 out of the 12 questions for junior students.

Quantitative results showed significant improvements in knowledge (*P*<.05) about antibiotic use, appropriate sneezing behaviors, and vaccinations for both age groups: junior (7-11 years) and senior (11-16 years).

In total, 26 student focus groups with 126 students were conducted, 10 junior (7-11 years) and 16 senior (11-16 years) with approximately 4 to 6 students per focus group. Researchers observed that students enjoyed playing Body Busters more, and they were keen to answer questions to gain more lives, whereas Stop the Spread was more difficult and on occasion required some researcher explanation. The mean enjoyment score for Body Busters was 8.4/10 for juniors and 7.2/10 for seniors; the mean enjoyment score for Stop the Spread was 6.2/10 for juniors and 5.1/10 for seniors (illustrated in Table 2).

### Qualitative Data

#### Reported Views of Body Busters Game

##### Body Busters Positive Perceptions: User Experience

Qualitative results from focus groups and open questionnaire responses for Body Busters were overall very positive with a few suggestions for improvement. Many students of both age groups reported positive perceptions of user experience; at least 1 participant in each focus group reported positive levels of enjoyment and nearly all other participants agreed. Many students wanted to play for longer as the game was very engaging, similar to Pac-Man, at the correct level of difficulty, and students reported that they had learned through the gaming experience:

I could be on there [Body Busters] for like the whole day or an hour, or actually 2 hours.

Junior student, Focus group 9

I feel like it was teaching us that antibiotics are to be used to kill a bacterial infection, but also that not all bacteria is harmful to the body.

Senior student, Focus group 5

I enjoyed the game play - collecting antibiotics and dodging the bad bacteria. The actual game was fun.

Senior student questionnaire response

I realised not all microbes are harmful.

Senior student questionnaire response

##### Body Busters Positive Perceptions: Game Functions

Many students of both age groups reported positive perceptions of the game functions, including game recovery aids, the pace of the game, and the game aesthetics. Some junior students also reported that they liked the microbe characters and the concept of being able to gain more lives. Some senior students also reported that they liked the different levels of the game and the useful pictorial instructions:

I liked getting more lives from the good bacteria and I liked collecting the antibiotics.

Junior student, Focus group 17

It was quite like, there was an equation, so the robot plus the circle equals health up, and that was quite a good way of formatting it without writing it out as paragraphs.

Senior student, Focus group 15

The game had good bacteria so you could regain your lives.

Senior student questionnaire response

##### Body Busters Negative Perceptions

A common negative theme for Body Busters was the slower pace of the game when users lost a life, reported by many junior and senior students:

I was cornered by two enemies and lost lots of lives-then I was really slow for the rest of the game. Becoming slow made it much less enjoyable.

Senior student questionnaire response

Some students suggested ways to modify this aspect of the game, including when the user loses a life, do not slow the avatar down but instead increase the speed or size of the harmful bacteria and viruses. Other students reported that the instructions could be improved by keeping the pictorial instructions but adding written instructions for clarity, and a few students suggested having a visible key for the different microbes in the game play:

Maybe...if you lose your health maybe there’s like a circle around the bacteria so that their like range gets bigger, but you’re still the same speed, or they get bigger themselves and you just increase the size of the map so two of them can go.

Senior student, Focus group 15

I think you should make it not go so slow and I think when you press start it should like count 1, 2, 3 go, so then you actually know where you are and you know where everything else is, you can just go off.

Junior students, Focus group 17

**Table 2:** Enjoyment scores for the e-Bug games (score out of 10).

Students	Body Busters	Stop the Spread
Junior	8.4	6.2
Senior	7.2	5.1
All students	7.6	5.5

#### Reported Views of Stop the Spread Game

##### Stop the Spread Positive Perceptions: User Experience

Some students in both age groups reported positive perceptions of user experience, including reporting an increase in knowledge about the spread of infection and the importance of vaccinations:

Vaccinations protect other people, so if you have a vaccination then you won’t get any colds and stuff, and then you’re protecting other people because you can’t pass anything on to them.

Junior student, Focus group 16

I think it teaches you that if you know you have a cold before you go to school...you have a tissue and you make sure you sneeze into the tissue and put it in the bin, so it’s safe, or if it’s too bad,...you might want to stay away as you’ll infect loads of people and make it even worse.

Senior student, Focus group 21

The learning points -very obvious how severely something can spread.

Senior student questionnaire response

In most junior focus groups, at least 1 student voiced an increase in understanding appropriate health behaviors, especially about sneezing, and most participants concurred with them. In some focus groups, students reported intent to change health behaviors:

It was teaching us that you should always use a tissue and put that in the bin.

Junior student, Focus group 16

It shows you what to not do, like if you feel like you’re going to sneeze and cough, do it into a tissue.

Junior student, Focus group 17

That we need to sneeze into a tissue not just sneeze out...Put it in the bin, don’t keep it in your coat pocket like I used to do.

Junior student, Focus group 19

##### Stop the Spread Positive Perceptions: Game Functions

Some students, junior and senior, enjoyed the fast pace of the game and different levels of difficulty, especially the vaccination levels. A few senior students reported on the game aesthetics: the look, appearance, and style of the game:

I liked how you could like vaccinate them, and everyone was dying, it kept on going then you had to get it really really quickly before anyone else got the illness to spread.

Junior student, Focus group 11

For me the vaccinations was one of the highlights that you had these certain people that were immune so you could just concentrate on one certain area where the clump of uninjected people were

Senior student, Focus group 15

I enjoyed the colours, so when it showed you they were red and then purple, that was helpful.

Senior student, Focus group 22

You could protect people by injecting them.

Senior student questionnaire response

##### Stop the Spread Negative Perceptions

A common negative theme of Stop the Spread was that the game was too difficult. In most focus groups, the majority of students felt the game was “too hard” because it was “too fast” and “too many children were sneezing at the same time.” Many students reported a lack of engagement to continue to play the game as they had negative emotions, such as feeling “stressed” and “annoyed”:

Oh gosh level 1 was fine, but as the levels went on I was like oh gosh how are we meant to do this now.

Junior student, Focus group 12

Maybe it was a little bit too hard.

Junior student, Focus group 18

It’s really fast, and it’s really hard.

Senior student, Focus group 2

In most focus groups, suggestions for improvements were provided, including slow the pace of the game down to make it easier, have fewer children sneezing at the same time, or slow the time down between students sneezing, include more levels of different difficulty such as an *easy* level or have a tutorial level, and make the instructions clearer and simpler:

The thing is that they all sneezed at the same time and I didn’t have enough time to put the tissue in the bin, so that is really a struggle.

Junior student, Focus group 16

Yeah too fast paced, maybe slow it down to start off with one person infected, and then do levels as you did with the Pac man one.

Senior student, Focus group 22

The instructions could have been clearer.

Senior student questionnaire response

Suggestions for game improvements and modifications from the qualitative focus groups and evaluation questions are summarized in Textboxes 1 and 2.

### Quantitative Data

Tables 3 and 4 show the *percentage correct before* the game intervention, *percentage correct after* the game intervention, and the *P* value for junior (7-11 years) and senior (11-15 years) school-aged students, respectively. Table 5 shows a comparison of baseline and postgaming knowledge between age groups.

**Suggestions for Body Busters game improvements from qualitative focus groups and evaluation questions.**

Body Busters

1. When the user loses a life, do not slow the avatar down but either:
  - increase the speed of harmful bacteria and viruses or
  - increase the size of harmful bacteria and viruses
2. Make instructions clearer
  - keep the pictorial instructions
  - add written instructions
3. Have a visible key for the different microbes in the game play
4. Add more levels in different areas of the body

**Suggestions for Stop the Spread game improvements from qualitative focus groups and evaluation questions.**

Stop the Spread

1. Slow the pace of the game down to make it easier
  - have fewer children sneezing at the same time
  - slow the time down between students sneezing
2. Include more levels of different difficulty
  - have an option for an *easy* level
  - have a tutorial or practice level
3. Make the instructions simpler and clearer

**Table 3:** Improvement scores by question for junior schools.

Question or statement	Correct before, %	Correct after, %	Odds ratio (95% CI)	*P* value
Which of these microbes causes coughs and colds?	35	29	0.57 (0.21-1.46)	.29
What is the best way to treat an infection with a virus?	44	47	1.25 (0.55-2.92)	.70
Antibiotics help cure colds	33	28	0.60 (0.23-1.46)	.31
Which of these infections could antibiotics be used to treat?	31	46	2.88 (1.24-7.43)	.01^a^
Most coughs and colds get better without antibiotics	59	68	2.22 (0.97-5.54)	.06^b^
All microbes are bad or harmful	78	84	1.50 (0.68-3.41)	.36
You cannot infect other people around you through coughs and sneezes	79	78	0.93 (0.42-2.07)	>.99
The more people are vaccinated, the more people are protected from that infection	60	72	3.00 (1.23-8.35)	.01^a^
By getting vaccinated, you can also protect others around you from infection	59	68	2.22 (0.97-5.54)	.06^b^
Antibiotics (list); kill good and bad bacteria	22	33	2.30 (1.05-5.41)	.04^a^
Vaccinations (list); protect us from catching and spreading diseases	79	78	0.75 (0.21-2.46)	.79
The best way to stop microbes in coughs and sneezes spreading is to (list)	61	71	4.00 (1.29-16.4)	.01^a^

^a^Significant at .05.

^b^Approaching significance at .06.

**Table 4:** Improvement scores by question for senior schools.

Question or statement	Correct before, %	Correct after, %	Odds ratio (95% CI)	*P* value
Which of these microbes causes coughs and colds?	47	46	0.88 (0.52-1.49)	.71
What is the best way to treat an infection with a virus?	46	46	1.06 (0.65-1.71)	.91
Antibiotics help cure colds	61	49	0.24 (0.11-0.45)	<.001^a^
Which of these infections could antibiotics be used to treat?	56	63	1.80 (1.08-3.06)	.02^a^
Most coughs and colds get better without antibiotics	67	73	1.79 (1.10-2.95)	.02^a^
All microbes are bad or harmful	95	92	0.40 (0.15-0.95)	.04^a^
You cannot infect other people around you through coughs and sneezes	92	90	0.72 (0.37-1.37)	.36
The more people are vaccinated, the more people are protected from that infection	84	89	1.89 (1.04-3.55)	.04^a^
By getting vaccinated, you can also protect others around you from infection	55	65	2.54 (1.56-4.26)	<.001^a^
Antibiotics (list); kill good and bad bacteria	36	46	3.12 (1.78-5.74)	<.001^a^
Vaccinations (list); protect us from catching and spreading diseases	93	94	1.50 (0.63-3.73)	.42
The best way to stop microbes in coughs and sneezes spreading is to (list)	79	83	1.94 (1.03-3.79)	.04^a^

^a^Significant at .05.

**Table 5:** Comparison between age groups of baseline and postgaming knowledge.

Question or statement	Baseline, %			Postgaming, %		
	Junior correct before	Senior correct before	Difference in knowledge	Junior correct after	Senior correct after	Difference in knowledge
1. Which of these microbes causes coughs and colds?	34	47	13^a^	29	46	17^a^
2. What is the best way to treat an infection with a virus?	44	46	2^a^	47	47	0^b^
3. Antibiotics help cure colds	33	61	28^a^	28	49	22^a^
4. Which of these infections could antibiotics be used to treat?	31	56	25^a^	46	63	17^a^
5. Most coughs and colds get better without antibiotics	59	67	8^a^	68	73	5^a^
6. All microbes are bad or harmful	78	95	17^a^	84	92	8^a^
7. You cannot infect other people around you through coughs and sneezes	79	92	13^a^	78	90	12^a^
8. The more people are vaccinated, the more people are protected from that infection	60	84	24^a^	72	89	17^a^
9. By getting vaccinated, you can also protect others around you from infection	59	55	4^b^	68	65	3^b^
10. Antibiotics (list); kill good and bad bacteria	22	36	14^a^	33	46	13^a^
11. Vaccinations (list); protect us from catching and spreading diseases	79	93	13^a^	78	94	17^a^
12. The best way to stop microbes in coughs and sneezes spreading is to (list)	61	79	18^a^	71	83	12^a^

^a^A higher knowledge in senior students.

^b^A higher knowledge in junior students.

### Junior Student Knowledge About Antibiotics and Vaccinations

Over 70% of junior students had high baseline knowledge of learning outcomes covered in questions 6, 7, and 11, and there was only a small nonsignificant increase in correct answers:

(6) All microbes are bad or harmful (78%-84%; *P*=.36)

(7) You cannot infect other people around you through coughs and sneezes. True or false (79%-78%; *P*>.99)

(11) Vaccinations protect us from catching and spreading diseases (79%-78%; *P*=.79).

Low baseline knowledge (<40% correct answers) was seen in questions 1, 3, 4, and 10:

(1) Which of these microbes causes coughs and colds? Bacteria or fungus or virus (34%-29%; *P*=.29)

(3) Antibiotics help cure colds? True or false (33%-28%; *P*=.31)

(4) Which of these infections could antibiotics be used to treat? Bacterial or viral or fungal (31%-46%; *P*=.01)

(10) Antibiotics: *kill good and bad bacteria* (22%-33%; *P*=.04).

Knowledge of the 7- to 11-year-old students significantly improved for 4 of the questions (4, 8, 10, and 12):

(4) Which of these infections could antibiotics be used to treat? Bacterial or viral or fungal (31%-46%; *P*=.01)

(8) The more people are vaccinated; the more people are protected from that infection. True or false (60%-72%; *P*=.01)

(10) Antibiotics: *kill good and bad bacteria* (22%-33%; *P*=.04)

(12) The best way to stop microbes in coughs and sneezes spreading is to: “catch coughs and sneezes in a tissue and throw the tissue away” (61%-71%; *P*=.01).

Knowledge improvement for 2 other questions (5 and 9) was approaching significance (*P*=.06):

(5) Most coughs and colds get better without antibiotics. True or false (59%-68%; *P*=.06)

(9) By getting vaccinated, you can also protect others around you from infection. True or false (59%-68%; *P*=.06).

**Figure 3:**
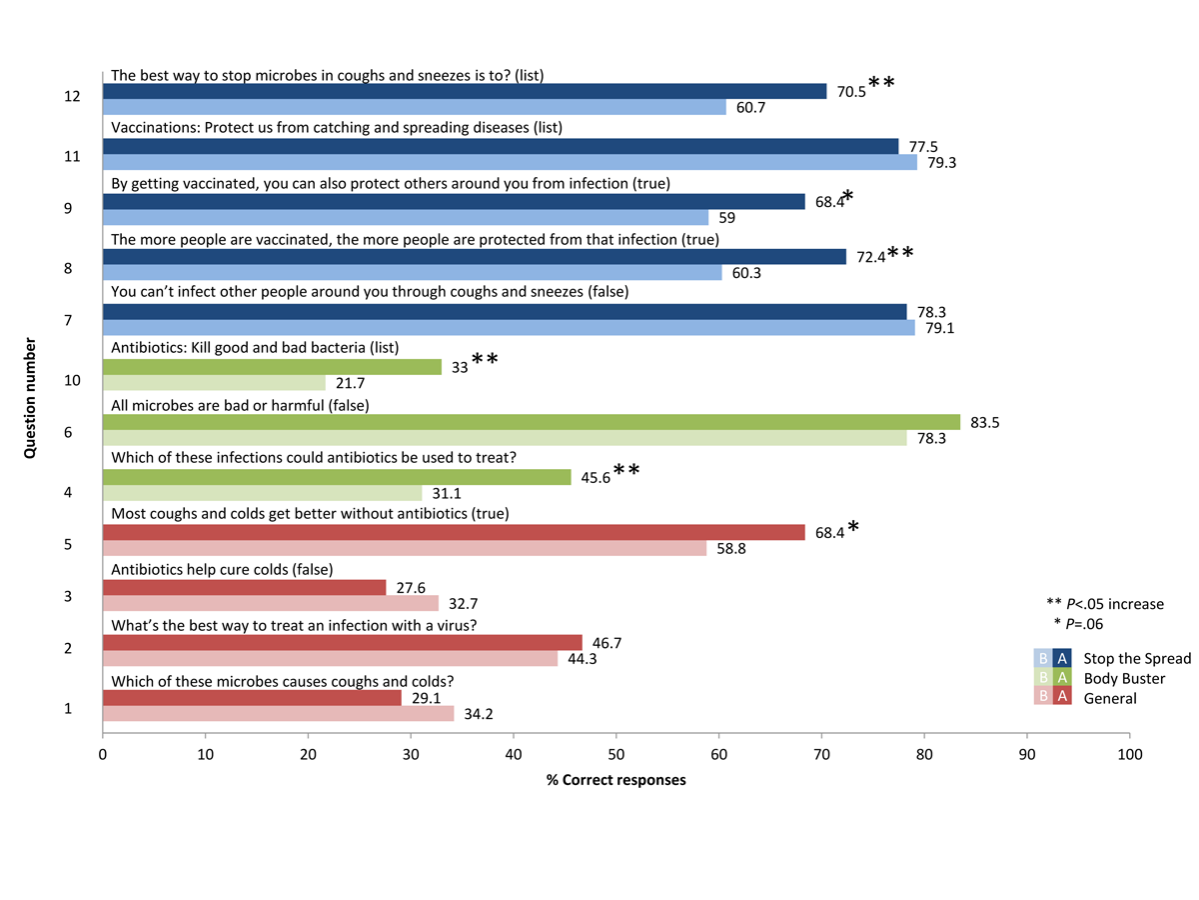
Percentage of junior students answering questions correctly before and after playing Body Busters and Stop the Spread Games.

Questions that saw the greatest improvement in knowledge for junior students were question 4 (31%-46%; *P*=.01), 8 (60%-72%; *P*=.01), and 10 (22%-33%; *P*=.04). Figure 3 provides before and after knowledge percentages and levels of significance.

There was no evidence of a significant knowledge change for questions 6, 7, and 11 or the 3 general questions (1, 2, and 3), which covered knowledge that could be indirectly gained from playing the 2 games.

### Senior Student Knowledge About Antibiotics and Vaccinations

Senior school students had greater baseline knowledge than junior students in 10 out of the 12 questions. Senior students had high baseline knowledge (>92% correct scores) to the same 3 questions as junior students (6, 7, and 11). In addition, the 2 questions (8 and 12) on vaccinations and sneezing behaviors had scores less than 70%. Senior students had significantly higher baseline knowledge scores for all bar 2 questions, so it was difficult to improve as much as the junior students. Low baseline knowledge (<40% correct scores) was seen in question 10 (Antibiotics [list] correct answer *Kill good and bad bacteria*).

**Figure 4:**
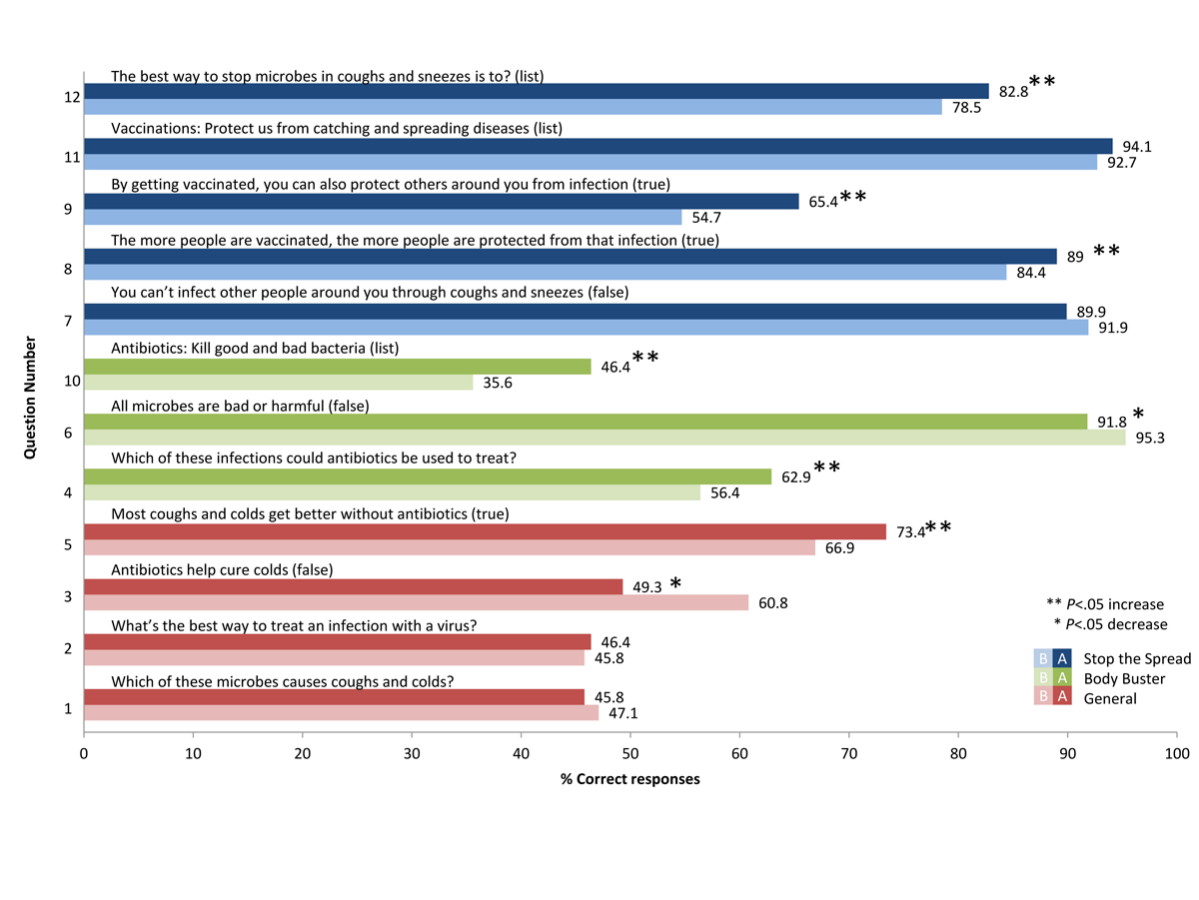
Percentage of senior students answering questions correctly before and after playing Body Busters and Stop the Spread games.

There were significant improvements in knowledge among senior students, with 6 out of the 12 questions showing a significant increase in the odds of students answering correctly (4, 5, 8, 9, 10, and 12), which were the same 6 questions that showed significant improvements among junior students. Questions that saw the greatest improvement in knowledge for senior students were questions 9 (By getting vaccinated, you can also protect others around you from infections; 10.7%) and 10 (Antibiotics kill good and bad bacteria; 10.8%). Figure 4 provides before and after knowledge percentages and levels of significance.

Question 6 (All microbes are bad or harmful; false), evaluating a learning outcome of Body Busters, and a general question 3 (Antibiotics help cure colds; false) showed a significant decrease in knowledge (95%-92% and 61%-49%, respectively).

There was no evidence of a significant knowledge change for questions 7 (You cannot infect other people around you through coughs and sneezes; false) and 11 (Vaccinations protect us from catching and spreading diseases), for which over 90% answered correctly before playing the games. Two other general questions, 1 (Which of these microbes causes coughs and colds?) and 2 (What is the best way to treat an infection with a virus?), did not see a significant change in knowledge.

## Discussion

### Principal Findings

This study indicates that playing the 2 e-Bug games had a significant (*P*<.05) positive effect on students’ knowledge on 6 out of the 12 questions:

1. Antibiotics are used to treat bacterial infections (question 4)
2. Antibiotics kill good and bad bacteria (question 10)
3. Most coughs and colds get better without antibiotics (junior *P*=.06; question 5)
4. The best way to stop microbes in coughs and sneezes spreading is to catch coughs and sneezes in a tissue and throw the tissue away (question 12)
5. The more people are vaccinated, the more people are protected from that infection (question 8)
6. By getting vaccinated, you can also protect others around you from infection (junior *P*=.06; question 9).

However, the games were indicated to have a detrimental effect on 2 true or false questions in the older students aged 11 to 16 years:

(3) Antibiotics help cure colds (false)

(6) All microbes are bad or harmful (false).

Suggestions for this detrimental effect include the following: question 3 was perhaps not obvious that viruses cause colds in either of the games, and question 6 had a very high baseline knowledge of 95%; therefore, it would have been difficult to see an improvement in knowledge. Modifications to the games will be required to address this detrimental effect, and developers should consider the age group of their target audience.

Another main finding of the study was the comparison between age groups of baseline knowledge and postintervention knowledge: junior (7-11 years) and senior (12-15 years). Baseline knowledge for senior students was higher than junior students on 11 out of the 12 questions; juniors scored 4.3% higher in question 9. Postintervention knowledge for senior students was higher than that for junior students in 10 out of the 12 questions; the 2 questions that were lower than juniors (2 and 9) were only lower by 0.3% and 4.3%, respectively. Positive knowledge change for juniors was greater in 9 out of the 12 questions compared with senior students, suggesting that the e-Bug games had a greater impact on junior student knowledge; the researchers therefore recommend that the e-Bug games should be targeted at junior school–aged children and should be further promoted to this age group.

The high baseline knowledge for senior students could be a reflection that the questions were too easy for older students and perhaps senior students obtained other learning from the games, which researchers could have picked up with different or more difficult questions.

Overall, both junior and senior students reported Body Busters to be more enjoyable than Stop the Spread on the Likert scale responses and thematic analysis of the focus group transcripts; this is supported by Google Analytics. During the academic year, September 1, 2016, to August 31, 2017, junior and senior students viewed Body Busters (8905 and 3814 views, respectively) more than Stop the Spread (6803 and 3027 views, respectively). During the same academic year, on average, junior and senior students played Body Busters (01:40 and 01:51 min, respectively) for longer than Stop the Spread (01:01 and 01:36 min, respectively). This may be because Body Busters was easier and more enjoyable; however, Stop the Spread lead to a greater improvement in knowledge, particularly about appropriate sneezing behaviors and vaccinations in this study. Suggestions for improvements on both games were provided by students, and the e-Bug team will consider the suggestions when making modifications for improvement.

### Strengths and Limitations

A mixed-method approach is a strength of the study; using both quantitative and qualitative methods of enquiry enables students’ knowledge to be measured and students’ views and intentions to be explored in some depth. The study is cross-sectional and representative of schools and students across the United Kingdom; a large number of students from a range of schools in different areas of the United Kingdom with different levels of deprivation were involved. This allows us to evaluate baseline knowledge about vaccinations and antibiotics in young people, the largest sample of this type. Baseline knowledge can be used to inform educational needs in different age groups within National Institute of Health and Care (NICE) recommendations. Qualitative focus groups enabled the exploration of a range of students’ views; it also brought synergism, snowballing of ideas, and stimulation of participants, which will assist in making improvements to the games.

The study evaluation allowed students to play each game in a classroom setting for only 5 min. This mimicked a real-life class setting where they would usually play Web-based games and discuss the games together, and 5 min is the usual length of time it takes to play 1 game. However, the 5 min of game play might not replicate the normal duration for game play; 5 min per game might not have been long enough for some students to gain the desired knowledge. For example, in Body Busters, viruses only appeared in level 3, and some students might have struggled to reach this level, which could partly explain any variation in percentage correct answers. The intervention in this study was used in isolation and perhaps the learning outcomes can be achieved better when the games are used as a tool to reinforce teaching about each topic in the classroom or in the home environment.

The questionnaires used in this study were based on questionnaires that have been used in previous e-Bug evaluations [2,3,16]. However, to eliminate any question style bias, future work with young students should use a questionnaire design that has the same format for each question, that is, all multiple-choice questions with 1 correct answer or all true or false questions to make it easier to understand for younger students.

Baseline knowledge was very high in senior students, especially about vaccinations and sneezing behaviors, so there is little need for improvements; however, modifications, including adding more levels to the games or adding extra learning outcomes, are required.

In the focus groups and evaluation questionnaires, junior students found it difficult to vocalize their thoughts beyond close-ended questions. Furthermore, many junior students found writing answers to the open responses on the evaluation form difficult to express their views, which was observed by researchers during data collection; however, data saturation was reached during the focus groups, suggesting no new themes would emerge.

### Comparison With Existing Literature

Improvements in students’ knowledge after the delivery of an e-Bug lesson [2,3,4,6] and the e-Bug Web-based games [16] have been well documented. Our research adds to the body of literature to support the value of the e-Bug resources and Web-based games in educating children on hygiene and antibiotic topics. Limited research has been conducted about school-aged children’s knowledge of antibiotics and vaccination topics in England. One e-Bug evaluation across 3 European countries found that junior- and senior-aged students in 1 county in England had high baseline knowledge about the spread of infection (68%-78%) and low levels of baseline knowledge about the treatment and prevention of infection (29%-34%) [6]; this is reflected in this study as students had greater baseline knowledge about vaccinations than antibiotics generally.

A previous evaluation [16] of an earlier version of Body Busters showed that it increased knowledge of antibiotics in children, created a flow-like state in players, and was enjoyed the most out of the 3 games evaluated. Suggestions for modifications to the game included the following: more information in the introductory text, make the difference between viruses and bacteria more obvious, and create a steady increase in difficulty level as the game progresses [16]. The changes suggested by Hale et al were made in 2015 and aided the enjoyment reported by students in this study. However, this study is a much larger evaluation including more student questionnaires and focus group responses from a wider student sample across the United Kingdom; therefore, this evaluation provides new evidence to support the importance of the e-Bug project, for future modifications to the games and for future e-Bug game developments.

Systematic reviews and meta-analyses of gamification, serious games, and apps about health topics found that they can have a positive impact on health and well-being in the general population [17-20]. Other gamification studies reported positive associations between gamification and school-aged knowledge in several health topics; a randomized controlled trial (RCT) of a serious game promoting oral health found a significant improvement in knowledge of children after playing the game compared with before playing the game [13]. Likewise, an RCT for a game educating children about asthma found an increase in knowledge and improved attitudes at postintervention and follow-up as compared with control [11]. This study adds to the literature to support the positive effect of serious games on knowledge and attitudes of school children on important health issues. A meta-analysis of serious games for healthy lifestyle promotion was found to be appealing to individuals regardless of age or gender, showing this could be an intervention suitable for a more general audience than just children [20].

Other antimicrobial games have been developed but very few have been evaluated and on such a large scale. Recently, there has been an increase in serious games about the topic of microbiology and antimicrobial resistance, such as the Longitude prize’s *Superbugs*, showing this is a rapidly growing area of serious games. An infection prevention gamification tool called Germ Defence, encouraging individuals of any age to pledge to wash their hands more often, has been evaluated as part of a large RCT [21]. The RCT with over 20,000 participants found that those who used the Germ Defence website had fewer colds, flu, and stomach upsets than those who had not seen the website [21], providing further evidence for the positive effect of gamification on knowledge and health behaviors.

### Implications for Future Research

Students had significant increases in knowledge about antibiotics, showing that e-Bug helps to reinforce the 2017 national antibiotics campaign *keep antibiotics working*. Students had significant increase in knowledge and behavioral intentions about appropriate sneezing behaviors, which directly support the 2013 national campaign *catch it, bin it, kill it*. Future national infection-related public health campaigns could link to the e-Bug games to encourage schools to use the games in their teaching and reinforce the campaigns.

Further research is required to investigate whether the knowledge gained from the e-Bug games is maintained or has changed future behavior. Additional qualitative research with teachers is needed to explore and understand how the e-Bug educational games can be used in a lesson to support learning.

e-Bug will continue to follow NICE guidance and work with educators and students to develop and promote resources for teaching children and young people about microbes, infection, and antibiotics in a fun and interactive way.

### Conclusions

Science pedagogy Web-based games, including the e-Bug games, have the potential to engage and excite children and young people about important public health topics and aid in the learning of knowledge. To increase gaming, the e-Bug games should be both fun and challenging.

This study shows that 2 e-Bug educational games, Body Busters and Stop the Spread, covering learning topics about microbes, infection prevention, and antibiotics, are valuable to school-aged children’s knowledge. Body Busters is greatly enjoyed by and engaged school-aged children; a few modifications about antibiotics that can kill good and bad bacteria are required to reinforce learning outcomes. Stop the Spread is enjoyed by school-aged children to a lesser degree and more modifications, including slowing the game down, are required to retain user engagement. However, learning outcomes are very well covered in Stop the Spread. Health commissioning schools should target and promote the Body Busters and Stop the Spread e-Bug games, especially toward junior students (aged 7-11 years), as they showed the greatest improvement in knowledge. Further levels with more learning outcomes will facilitate increased learning in older students (aged 12-15 years).

## Appendix

Multimedia Appendix 1 Student questionnaires.

## Abbreviations

NICE: National Institute of Health and Care
PHE: Public Health England
RCT: randomized controlled trial
