# Supplementary material for: Young People’s Knowledge of Antibiotics and Vaccinations and Increasing This Knowledge Through Gaming: Mixed-Methods Study Using e-Bug
Source: JMIR Serious Games. 2019 Feb 1;7(1):e10915. doi: 10.2196/10915 (PMC6376338; doi:10.2196/10915)
Supplement: Multimedia Appendix 1 [file games_v7i1e10915_app1.pdf]

Questionnaire 1: before knowledge

Name:	Age:

e-Bug game evaluation questionnaire

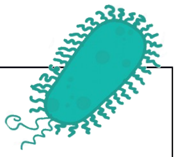

**1. Which of these microbes causes coughs and colds?**	
Bacteria	
Fungus	
Virus	X

**3. Antibiotics help cure colds?**	
True	
False	X

**2. What's the <u>best</u> way to treat an infection with a virus?**	
Eating lots	
Water and bed rest	X
Antibiotics	
Exercise	

**4. Which of these infections could antibiotics be used to treat?**	
Bacterial	X
Viral	
Fungal	

	**TRUE**	**FALSE**	**DON'T KNOW**
**5. Most coughs and colds get better without antibiotics.**	X		
**6. All microbes are bad/harmful.**		X	
**7. You can't infect other people around you through coughs and sneezes.**		X	
**8. The more people are vaccinated; the more people are protected from that infection.**	X		
**9. By getting vaccinated, you can also protect others around you from infection.**	X		

Please tick one box for each question √

**10. Antibiotics…**	
a) Are good at killing viruses	
b) Make your smarter	
c) Kill good and bad bacteria	X

**11. Vaccinations…**	
a) Are bad for us	
b) Do not protect you from viruses	
c) Protect us from catching and spreading diseases	X

**12. The best way to stop microbes in coughs and sneezes spreading is to:**	
a) Catch coughs and sneezes in a tissue and throw the tissue away	X
b) Cough and sneeze into your hand	
c) Take antibiotics	
d) Cough and sneeze into your sleeve	

Questionnaire 2: after knowledge:

Name:	Age:

e-Bug game evaluation questionnaire

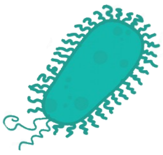

1. **Which of these microbes causes coughs and colds?**	
Bacteria	
Fungus	
Virus	X

2. **What's the best way to treat an infection with a virus?**	
Eating lots	
Water and bed rest	X
Antibiotics	
Exercise	

3. **Antibiotics help cure colds?**	
True	
False	X

**4. Which of these infections could antibiotics be used to treat?**	
Bacterial	X
Viral	
Fungal	

	**TRUE**	**FALSE**	**DON'T KNOW**
**5. Most coughs and colds get better without antibiotics.**	X		
**6. All microbes are bad/harmful.**		X	
**7. You can't infect other people around you through coughs and sneezes.**		X	
**8. The more people are vaccinated; the more people are protected from that infection.**	X		
**9. By getting vaccinated, you can also protect others around you from infection.**	X		

Please tick one box for each question √

**10. Antibiotics…**	
a) Are good at killing viruses	
b) Make your smarter	
c) Kill good and bad bacteria	X

**11. Vaccinations…**	
a) Are bad for us	
b) Do not protect you from viruses	
c) Protect us from catching and spreading diseases	X

**12. The best way to stop microbes in coughs and sneezes spreading is to:**	
a) Catch coughs and sneezes in a tissue and throw the tissue away	X
b) Cough and sneeze into your hand	
c) Take antibiotics	
d) Cough and sneeze into your sleeve	

Please answer the following questions on the enjoyability of the games:

1. How enjoyable was the Body Busters game to play? Please circle *(1 is not enjoyable, 10 is very enjoyable)*

   1 2 3 4 5 6 7 8 9 10

2. What was your favourite part of Body Busters?

………
…….

3. What was your least favourite part of the game?

………
…….

4. How enjoyable was the Stop the Spread game to play? Please circle *(1 is not enjoyable, 10 is very enjoyable)*

1 2 3 4 5 6 7 8 9 10

5. What was your favourite part of Stop the Spread?

………
…….

6. What was your least favourite part of the game?

………
…….

7. Any other comments on the activities you have done today?

………
………
……….
